# MicroRNA-136-3p inhibits glioma tumorigenesis in vitro and in vivo by targeting KLF7

**DOI:** 10.1186/s12957-020-01949-x

**Published:** 2020-07-16

**Authors:** Yanwu Xu

**Affiliations:** Neurosurgery Department, People’s Hospital of Lanling County, Linyi, Shandong province, 277700 China

**Keywords:** miR-136-3p, KLF7, Glioma, Xenograft tumor model

## Abstract

**Background:**

Malignant brain tumors have been a serious threat to human health worldwide. This study aims to investigate the role of miR-136-3p in glioma development.

**Methods:**

Hematoxylin-eosin staining (H&E) staining was used to determine the pathologic alterations of glioma tissues. Quantitative real-time PCR (qRT-PCR) analysis and GEO2R analysis was performed to examine the expression of miRNAs and genes. Western blot was applied to detect the protein expression. Cell counting kit-8 (CCK-8) and colony formation were used to analyze the glioma cell growth. Trans-well assay was used to determine the cell migration. Annexin V-FITC/PI staining was conducted to determine the cell apoptosis of transfected glioma cells. The dual-luciferase reporter assay was carried out to confirm the binding sites of miR-136-3p on 3′ untranslated regions (3′ UTR) of Kruppel-like factor 7 (KLF7). Tumor-bearing experiment in nude mice was performed to comprehensively investigate the role of miR-136-3p/KLF7 axis in gliomas.

**Results:**

Firstly, the results showed that miR-136-3p was decreased in glioma tissues compared with adjacent tissues. Overexpression of miR-136-3p significantly inhibited cell growth of LN-229 and U251 by decreasing expression of Cyclin A1 and PCNA (proliferating cell nuclear antigen), and it suppressed glioma cell migration by downregulating N-cadherin and elevating E-cadherin levels, and it also promotes glioma cell apoptosis by promoting Bcl2-associated X (Bax) expression but suppressing Bcl-2 expression. Furthermore, we observed that KLF7 was a direct target of miR-136-3p, and KLF7 was negatively regulated by miR-136-3p in glioma cells. Finally, overexpression of KLF7 partly blocked miR-136-3p-induced inhibition of tumor growth in vitro and in vivo.

**Conclusions:**

Targeting miR-136-3p/KLF7 axis might be a novel manner to counter against gliomas.

## Background

Glioma presents one of the most common malignant cancers [1, 2]. Although multiple therapies, including surgery, radiotherapy, and chemotherapy, have been developed for therapeutic treatment of glioma, the prognosis remains poor and the survival rate of 5 years no more than 10% due to its heterogeneous characteristics [1, 3]. Up to now, few biological risk factors and effective therapeutic targets have been identified to better glioma treatment [4–6]. Thus, it is vital to find novel prognostic and therapeutic targets for glioma patients.

MicroRNAs (miRNAs) are small non-coding RNAs with length of 18-22 nucleotides. It has been reported that binding to 3′ untranslated region (3′ UTR) of targeted mRNA is a chief manner for miRNAs exerting their function in diseases [7–9]. Increasing evidences support that a series of miRNAs acts as an essential role in tumorigenesis including cell proliferation, migration, apoptosis, and angiogenesis [10–13]. For example, miR-622 levels predict poor prognosis and it exhibits functions by targeting zinc finger E-box binding homeobox 2 (ZEB2) in glioma [6]. MiR-532-5p inhibits glioma cell proliferation by targeting colony-stimulating factor 1 (CSF1) [14]. MiR-199a suppresses tumor growth and ameliorates chemoresistance via targeting K-RAS via protein kinase B α (namely AKT1) and extracellular-regulated protein kinases (ERK) pathways [15]. In this study, we selected to investigate the role of miR-136-3p in gliomas by performing GEO2R analysis of microarray GSE103228, and this bioinformatic data showed that miR-136-3p was decreased in glioma tissues. In the previous studies, miR-136-3p inhibits cell growth and metastasis by targeting solute carrier family 7 member 5 (SLC7A5) and ADAM metallopeptidase domain 9 (ADAM9) in thyroid cancer [16], suggesting that miR-136-3p might function as a tumor suppressor in cancers including gliomas. Similarly, miR-136-5p could enhance glioma cell apoptosis via targeting MTDH (Metadherin) and Bcl-2 [17]. Based on the previous studies, we can conclude that the role of miR-136-3p remains unknown in gliomas, and its roles in gliomas need to be further investigated.

To explore the potential molecular mechanisms of miR-136-3p in gliomas, we predicted its targets using *TargetScan database*. Integration of this bioinformatic analysis with the previous studies, we observed that KLF7 was a direct target of miR-136-3p in gliomas. In the previous studies, KLF3, targeted by miR-450b-3p and miR-185, often severs as an oncogene in gastric cancer [18] and non-small cell lung cancer respectively [19]. And KLF7, regulated by Linc00669/miR-193a axis, may also promote progression of non-small cell lung cancer [20]. Importantly, KLF7 can enhance glioma progression by transcriptionally activating argininosuccinate lyase [21]. Consequently, we can conclude that KLF7 is an oncogene in cancers, such as glioma. Herein, we investigated the role of miR-136-3p/KLF7 axis in glioma cell proliferation, migration, and apoptosis, and we also explored the potential molecular mechanisms. The findings showed that overexpression of miR-136-3p might provide a novel therapeutic target for glioma development.

## Methods and materials

### Collection of cancer tissues

In this study, adjacent tissues and glioma tissues were collected from 41 patients in Shandong Provincial Lanling People’s Hospital. The clinical processes were approved by the ethics committee of this Hospital. None of the patients had received treatment before surgery. These collected tissues were put in liquid nitrogen.

### Hematoxylin-eosin (H&E) staining

We fixed the adjacent tissues and glioma tissues in 4% paraformaldehyde (Sigma, Shanghai, China). We stained the clinical tissues with hematoxylin and eosin (Sigma) as described previously [1].

### Cell culture

The U251 and LN-229 were bought from ATCC (American Type Culture Collection, Manassas, VA, USA), and they were cultured in Dulbecco’s Modified Eagle Medium (DMEM) basic media (Thermo Fisher Scientific, Waltham, Massachusetts, USA), and the media contained 10% fetal bovine serum, and 100 mg/mL streptomycin, and 100 U/mL penicillin (Thermo Fisher Scientific). They were cultured in a 37 °C sterile incubator with 5% CO_2_.

### Plasmids, small interfering RNAs, and lenti-viruses

All the plasmids and RNA fragments were purchased from Sangon (Shanghai, China). These DNAs or RNAs were transfected into glioma cells with Lipofectamine 3000 (Thermo Fisher Scientific). The pcDNA3.1 or lenti-vector was used as an empty vector, and pcDNA-KLF7 plasmid, as well as lenti-KLF7, contained a full length of KLF7 complementary DNA, were employed to overexpress KLF7 in glioma cells. MiR-NC (5′-UCU CCG AAC GUG UCA CGU U-3′) or lenti-miR-NC was applied as a negative control, and miR-136-3p mimic (5′-CAU CAU CGU CUC AAA UGA GUC U-3′) or lenti-miR-136-3p was used to overexpress miR-136-3p, and anti-miR-136-3p (5′-AGA CUC AUU UGA GAC GAU GAU G-3′) was applied to knock miR-136-3p down. On the other hand, pmirGLO-KLF7-3′ untranslated region (UTR)-WT (wide type) or −Mut (mutant) were constructed by Sangon used for determining the interaction between miR-136-3p and KLF7, and mutant binding sites were synthesized by Sangon.

### Quantitative real-time PCR

We extracted total RNA using TRIzol reagent (Thermo Fisher Scientific). The purified RNA (1 μg) was used to produce complementary DNA (cDNA) using a kit for reverse transcription (Thermo Fisher Scientific). Then, SYBR Green Kit, from Thermo Fisher Scientific, was used to determine the expression of miRNAs and mRNAs. The U6 or β-actin was considered as an internal control of miR-136-3p or mRNAs, respectively. The primers were shown below. Cyclin A1: forward, 5′-CTC TTA ACC GCG ATC CTC C-3′, reverse, 5′-CCA TCC CAA GTG ACG AGC A-3′. PCNA: forward, 5′-CTC TTC CCT TAC GCA AGT CTC A-3′, reverse, 5′-GTG CCT CCA ACA CCT TCT TG-3′. E-cadherin: forward, 5′-AAC AGC AAA GGG CTT GGA TTT TG-3′, reverse, 5′-CAG CCA GTT GGC AGT GTC TC-3′. N-cadherin: forward, 5′-TGC ATG AAG GAC AGC CTC TT-3′, reverse, 5′-TGG GTC TCT TTG TCT TGG GC-3′. Bax: forward, 5′-GGG TTG TCG CCC TTT TCT AC-3′, reverse, 5′-AGT CGC TTC AGT GAC TCG G-3′. Bcl-2: forward, 5′-ATG CGG CCT CTG TTT GAT TTC-3′, reverse, 5′-GCA GGC ATG TTG ACT TCA CTT-3′. KLF7: forward, 5′- -3′, reverse, 5′- -3′. β-actin: forward, 5′-GCT CAC CAT GGA TGA TGA TAT CGC-3′, reverse, 5′-TAG GAA TCC TTC TGA CCC ATG CC-3′. MiR-136-3p: forward, 5′-CAU CAU CGU CUC AAA U-3′, reverse, 5′-GTG CAG GGT CCG AGG T-3′. U6: forward, 5′-TGC GGG TGC TCG CTT CGG CAG C-3′, reverse, 5′-GTG CAG GGT CCG AGG T-3′. These used sequences of primers were bought from GeneScript (Nanjing, China). Finally, the relative level of miRNAs and mRNAs was calculated according to the 2^−ΔΔCt^ methods. The experiments were repeated for three times independently, and 3-well was prepared for each sample.

### Western blot

The supernatant, from lysed cancer cells or tissues, was used for determining the protein expression in this study using radio-immunoprecipitation assay (RIPA) lysate (Thermo Fisher Scientific). The lysates were boiled at 98 °C for 10 min. Then, 60 μg proteins were added on sodium dodecyl sulfate (SDS)-polyacrylamide gel electrophoresis (SDS-PAGE) (Sangon). Afterward, these gels were transferred to 0.22 nm polyvinylidene difluoride (PVDF) membranes for 60 min (Bio-Rad, Hercules, CA, USA). Then, PVDF membranes were blocked with 2% phosphate buffer saline (PBS)-diluted bovine serum albumin (BSA) were incubated with the primary antibody at 4 °C for 8 h. The protein bands were captured by using Tanon 4600SF system (Tanon, Shanghai, China) after coating with horseradish peroxidase (HRP)-conjugated goat-anti-mouse or goat anti-rabbit secondary antibody (Thermo Fisher Scientific). The anti-KLF7 and anti-β-actin were bought from Bioworld Technology (Nanjing, China). The experiments were carried out for three times independently.

### Cell counting kit-8 (CCK-8) assay

In our study, the cell proliferation of LN-229 or U251 was assessed by performing CCK-8 assay (Sigma). Firstly, 3 × 10^3^ cells/well was prepared in 96-well plates (Corning, Shanghai, China). After 48 h transfection with miRNAs or plasmids, the OD value was measured in the microplate reader (Thermo Fisher Scientific). The experiments were conducted for three times independently, and 5-well was prepared for each group.

### Colony formation assay

Firstly, 2000 cells/well was plated into a 6-well plate (Corning). After 48 h transfection with miRNAs or plasmids, the cells (LN-229 or U251) were maintained in normal fresh media. Next, the media was replaced every 3 days. Post 14 days incubation, the visible colonies were stained with 0.1% crystal violet (Sigma) after fixation with methanol for 1 h, and they were captured using a microscopy (Leica, WETZLAR, German). The experiments were conducted for three times independently, and 5-well was prepared for each group.

### Trans-well assay

Here, we put 1.0 × 10^5^ cells per well into the top-chambers of multi-well plates (Corning). Then, we added 10% FBS-contained DMEM into the down chambers. Post 16 h incubation, the migrated cells were stained with crystal violet after fixation with 4% paraformaldehyde. In the end, we observed the migrated cells with a microscopy under × 200 magnification (Leica). The experiments were carried out for three times independently, and 5-well was prepared for each group.

### Annexin-V/fluorescein isothiocyanate (FITC) and propidium iodide (PI) staining

The cancer cells were seeded in 24-well plates, and they were harvested at 80 g for 5 min post 48 h transfection. Afterward, the LN-229 or U251 was stained with an Annexin V-FITC/PI kit (Solaribio, Beijing, China) in the dark following the manufacturer’s instruction. Finally, the cell apoptosis rate of cancer cells was determined on Bio-Rad S3e flow cytometry (Hercules, CA, USA). The experiments were conducted for three times independently, and 3-well was prepared for each sample.

### Dual-luciferase reporter assay

In this study, 3 × 10^4^ cells (LN-229 or U251) was seeded in 24-well plates. The cells were co-transfected with pmirGLO-KLF7-3′ UTR-WT (wide type), pmirGLO-KLF7-3′ UTR-mut (mutant), and miR-NC (negative control), miR-136-3p mimic, and anti-miR-136-3p. Post 48 h transfection, we analyzed the luciferase activity using dual-luciferase assay kit (Promega, Madison, USA). The experiments were performed for three times independently, and 5-well was prepared for each sample.

### Animal experiments

The LN-229 cells were collected, and 6 × 10^6^ cells were planted into 5-week-old BALB/c nude mice. The mice were bought from the Model Animal Research Center of Nanjing University (Nanjing, China). At the initiation of tumor growth (on the third day), the lenti-viruses were injected into tumors, and this injection was repeated every 6 days. These animal experiments were approved by the ethics committee of Shandong Provincial Lanling People’s Hospital. Afterward, the tumor volume was measured using the formula (length × width^2^)/2 at the indicated time. Finally, the tumors were captured and weighted.

### Data analysis

The data here were indicated as the mean ± standard deviation (S.D.). A *p* value < 0.05 was statistically significant. All the showed data were analyzed statistically using GraphPad Prism 6 (GraphPad Software, CA, USA). The statistical significance was examined by Two-tailed Student’s *t* test for two groups comparisons and one-way analysis of variance (ANOVA) test with post hoc analysis contrasts for multi-groups comparisons.

## Results

### MiR-136-3p is elevated in glioma tissues

**Fig. 1:**
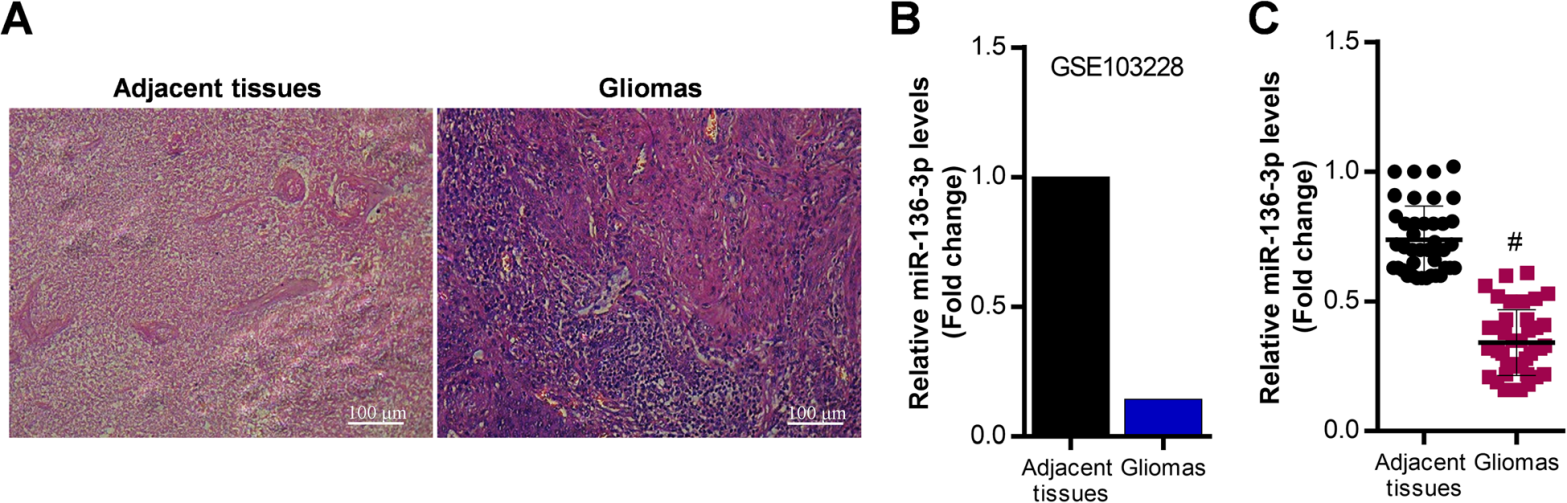
MiR-136-3p is up-regulated in glioma tissues. **a** H & E staining of pathologic alterations of glioma tissues. **b** GEO2R analysis of miR-136-3p in microarray GSE103228. **c** qRT-PCR analysis of miR-136-3p in adjacent tissues and in glioma tissues; #*p* < 0.01, compared with adjacent tissues

To investigate the role of miR-136-3p in glioma development, we collected tumors from patients with glioma in the hospital. The clinical characteristics of 41 patients were showed in Table 1. The H&E staining showed that glioma tissues showed significant pathologic alterations compared with adjacent tissues (Fig. 1a). GEO2R analysis and qRT-PCR analysis revealed that miR-136-3p was significantly decreased in glioma tissues compared with adjacent tissues (Fig. 1b, c).

**Table 1:** Clinical characteristics of 41 patients

Clinicopathologic feature	***n*** (%)	miR-136-3p			***p*** value
		High	Low	mean ± SEM	
**Age**					*p* = 0.3603
≤ 40	19 (46.34%)	10	9	1.89 ± 0.18	
≥ 40	22 (53.66%)	10	12	1.92 ± 0.17	
**Gender**					*p* = 0.2104
Female	18 (43.90%)	8	10	1.91 ± 0.19	
Male	24 (56.10%)	13	11	1.79 ± 0.18	
**Surgery**					*p* = 0.4472
Biopsy	7 (17.07%)	4	3	2.03 ± 0.20	
Partial resection	14 (34.15%)	5	9	2.15 ± 0.18	
Gross total resection	20 (48.78%)	9	11	2.07 ± 0.21	
**WHO grade** (%)					*p* < 0.0001
ІІ	8 (30.00%)	2	6	2.91 ± 0.26	
ІІІ	12 (33.33%)	4	8	2.05 ± 0.21	
IV	21 (36.67%)	5	17	1.68 ± 0.17	
**Low expression**	18 (43.90%)	13	5	4.19 ± 0.41	*p* < 0.0001
**High expression**	23 (56.10%)	4	19	1.98 ± 0.22	
**Tumor size**					*p* = 0.3514
< 4 cm	16 (39.02%)	7	8	2.18 ± 0.21	
≥ 4 cm	25 (60.98%)	11	14	2.24 ± 0.23	

### Overexpression of miR-136-3p suppresses glioma cell growth and migration

**Fig. 2:**
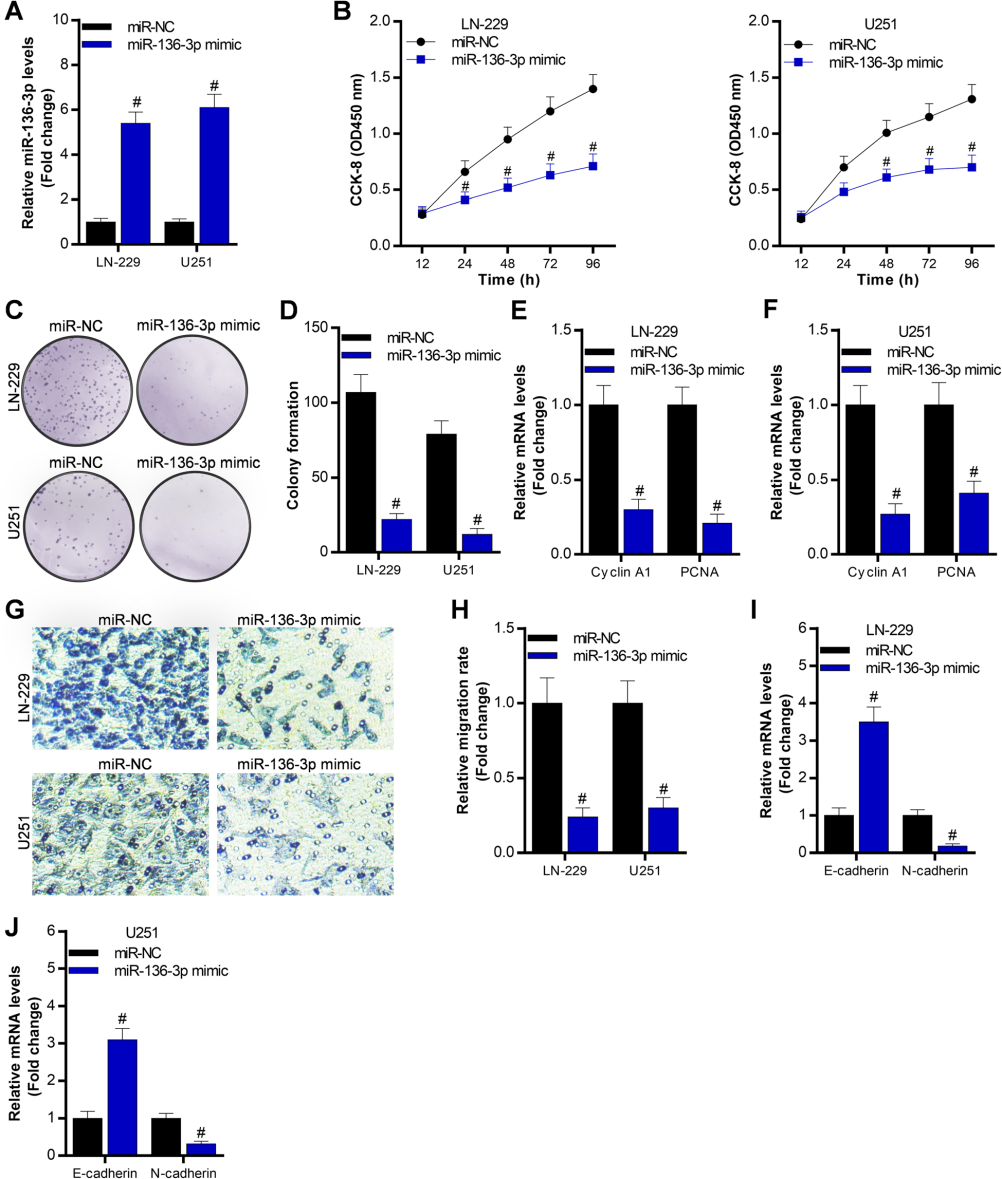
Overexpression of miR-136-3p inhibits glioma cell proliferation and migration in LN-229 and U251 cells. **a** qRT-PCR analysis of miR-136-3p in LN-229 and U251 cells post 48 h transfection with miR-NC or miR-136-3p mimic; #*p* < 0.01, compared with miR-NC. **b** CCK-8 assay determination of proliferation of LN-229 and U251 cells after transfection with miR-NC or miR-136-3p mimic at the indicated time; #*p* < 0.01, compared with miR-NC. **c**, **d** Soft-agar colony formation assay analysis of glioma cell growth after 15 days transfection with miR-NC or miR-136-3p mimic; #*p* < 0.01, compared with miR-NC. **e**, **f** qRT-PCR analysis of cell growth-associated genes, including Cyclin A1 and PCNA, in LN-229 and U251 cells after 48 h transfection with miR-NC or miR-136-3p mimic; #*p* < 0.01, compared with miR-NC. **g**, **h** Trans-well assay analysis of migration ability of transfected LN-229 and U251 cells with miR-NC or miR-136-3p mimic; #*p* < 0.01, compared with miR-NC. **i**, **j** qRT-PCR analysis of cell migration-associated genes, including E-cadherin and N-cadherin, in LN-229 and U251 cells after 48 h transfection with miR-NC or miR-136-3p mimic; #*p* < 0.01, compared with miR-NC

Here, we overexpressed miR-136-3p in LN-229 and U251 to detect the glioma cell growth and migration. The data showed that miR-136-3p mimics efficiently overexpressed miR-136-3p in LN-229 and U251 cells (Fig. 2a). The CCK-8 assay told that overexpression of miR-136-3p significantly inhibited glioma cell proliferation (Fig. 2b). Soft-agar colony formation assay analysis demonstrated that overexpression of miR-136-3p obviously repressed glioma cell colony formation of LN-229 and U251 cells (Fig. 2c, d). Meanwhile, overexpression of miR-136-3p significantly downregulated cell growth-associated gene expression, including Cyclin A1 and PCNA in LN-229 and U251 cells (Fig. 2e, f). Finally, trans-well assay demonstrated that overexpression of miR-136-3p markedly decreased the migration of LN-229 and U251 (Fig. 2g, h). Finally, we found that overexpression of miR-136-3p elevated expression of migration-associated gene E-cadherin, and it reduced N-cadherin levels in LN-229 and U251.

### Overexpression of miR-136-3p promotes glioma cell apoptosis

**Fig. 3:**
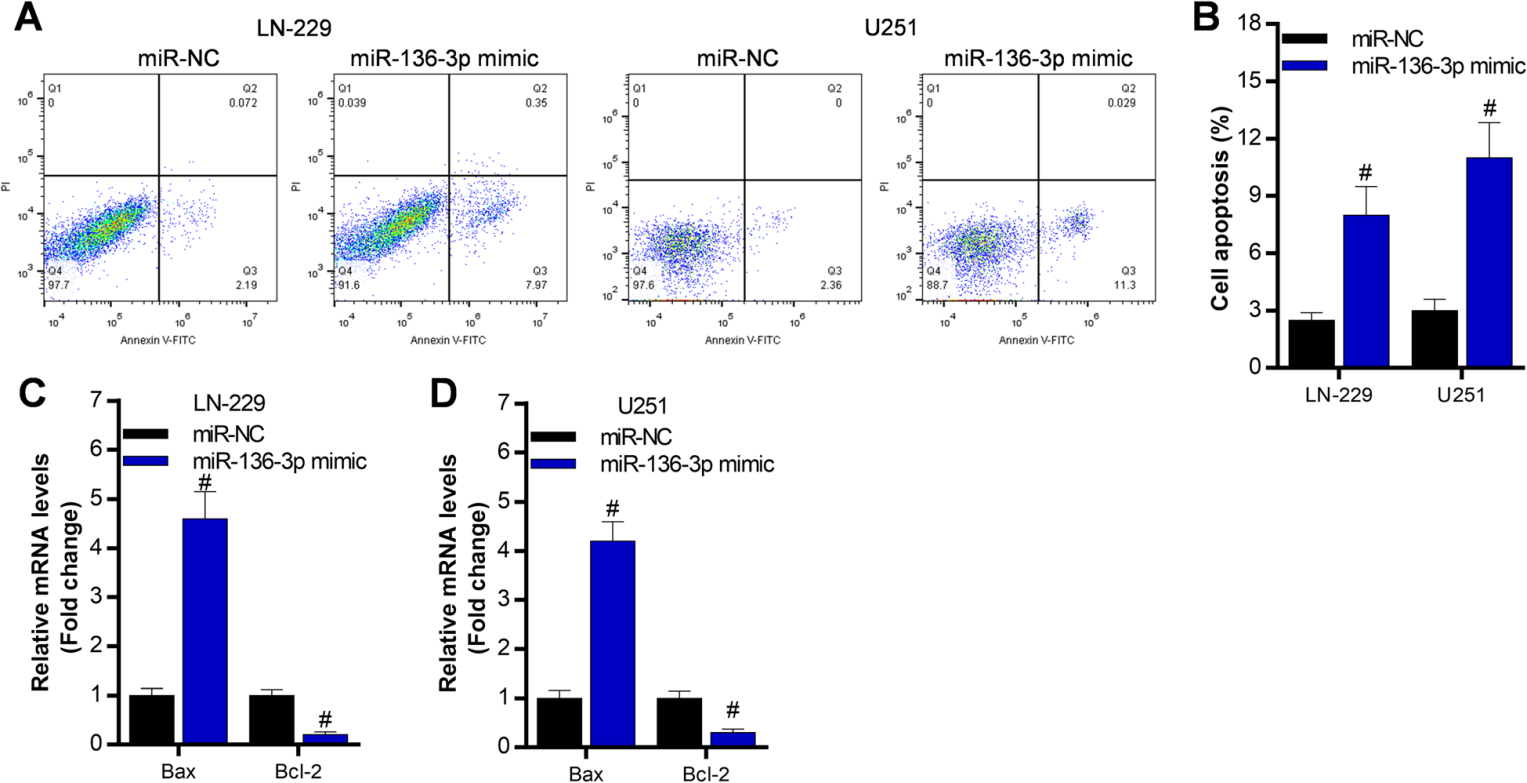
Overexpression of miR-136-3p increases cell apoptosis LN-229 and U251 cells. **a**, **b** Annexin V-FITC analysis of cell apoptosis in LN-229 and U251 after 48 h transfection with miR-NC or miR-136-3p mimic; #*p* < 0.01, compared with miR-NC. **c**, **d** qRT-PCR analysis of cell apoptosis-associated genes, including Bax and Bcl-2, in LN-229 and U251 cells after 48 h transfection with miR-NC or miR-136-3p mimic; #*p* < 0.01, compared with miR-NC

To determine the effect of miR-136-3p on glioma cell apoptosis, we conducted annexin V-FITC/PI staining. The data exhibited that overexpression of miR-136-3p significantly promotes apoptosis of LN-229 and U251 cells (Fig. 3a, b). Consistently, qRT-PCR demonstrated that overexpression of miR-136-3p elevated Bax expression, and inhibited Bcl-2 expression in LN-229 and U251 cells (Fig. 3c).

### KLF7 is a direct target of miR-136-3p in glioma cells

**Fig. 4:**
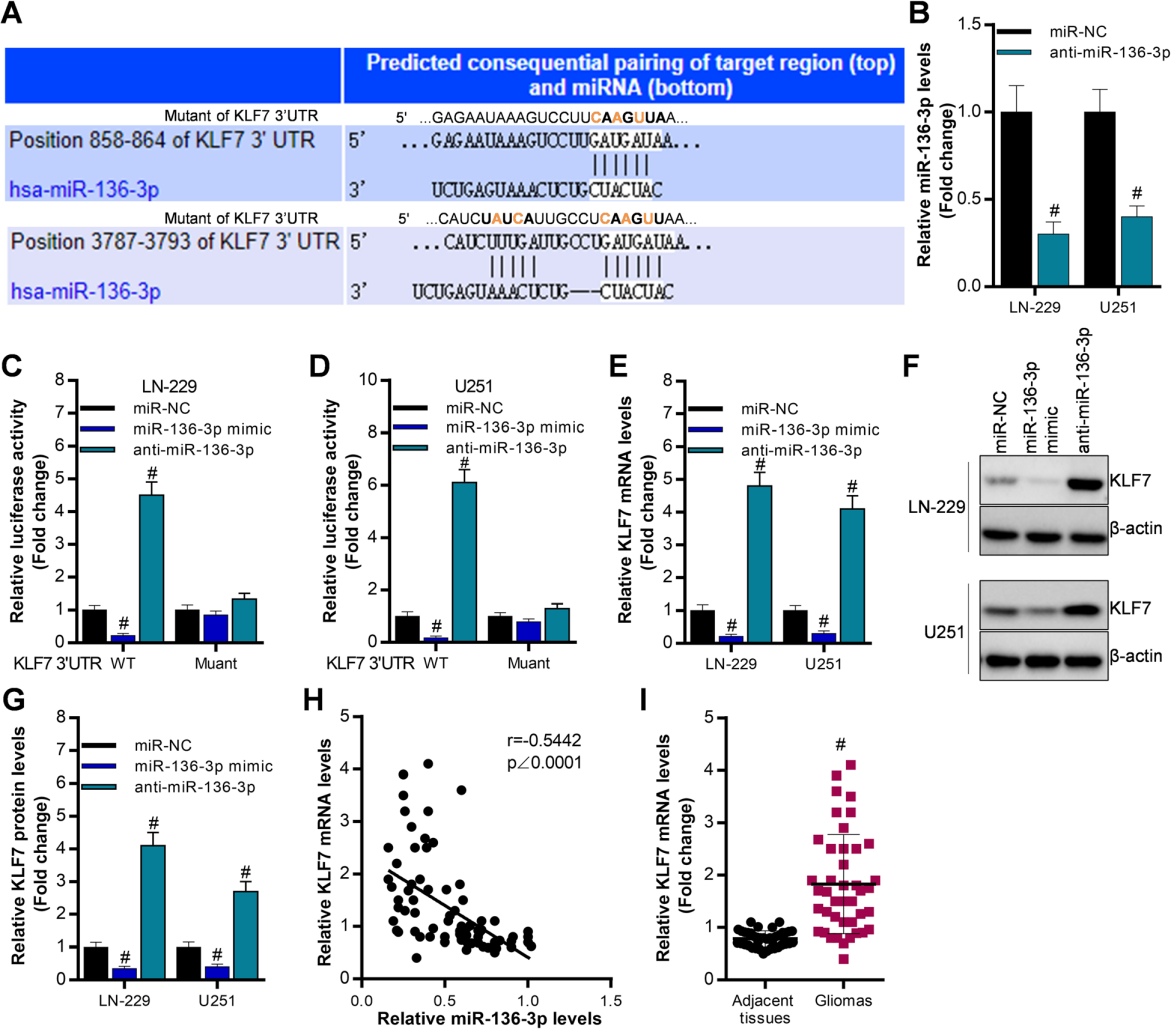
KLF7 is a downstream target of miR-136-3p. **a** The predicted binding sites of miR-136-3p on KLF7 3′ UTR using *TargetScan database*. **b** qRT-PCR analysis of miR-136-3p post 48 h transfection in LN-229 and U251 cells, #*p* < 0.01, compared with miR-NC. **c**, **d** Luciferase reporter assay determination of luciferase activity of KLF7 3′ UTR after 48 h transfection with miR-NC, miR-136-3p mimic, or anti-miR-136-3p in LN-229 and U251, #*p* < 0.01, compared with miR-NC. **e** qRT-PCR analysis of miR-136-3p post 48 h transfection with miR-NC, miR-136-3p mimic, or anti-miR-136-3p in LN-229 and U251, #*p* < 0.01, compared with miR-NC. **f**, **g** Western blot analysis of KLF7 expression after 48 h transfection with miR-NC, miR-136-3p mimic, or anti-miR-136-3p in LN-229 and U251, #*p* < 0.01, compared with miR-NC. **h** The correlation of miR-136-3p and KLF7 in glioma tissues using Graphpad prism. **i** qRT-PCR analysis of KLF7 in adjacent tissues and in glioma tissues; #*p* < 0.01, compared with adjacent tissues

To investigate the potential molecular mechanism of miR-136-3p in gliomas, we predicted the downstream targets of miR-136-3p using *TargetScan tools*. Base on comprehensive consideration of both the bioinformatic prediction and the previous studies [19–22], we can conclude that KLF7 is an oncogene in glioma or other cancers. Thus, we selected KLF as the target of miR-136-3p in glioma (Fig. 4a). Luciferase reporter assay expressed that overexpression of miR-136-3p significantly inhibited luciferase activity of KLF7 3′ UTR, which was upregulated by knocking miR-136-3p down in LN-229 and U251 (Fig. 4c, d). Then, qRT-PCR analysis and western blot analysis revealed that miR-136-3p negatively modulated KLF7 expression (Fig. 4e, f, g). Moreover, the miR-136-3p expression is negatively associated with KLF7 levels in clinical samples (Fig. 4h). Additionally, KLF7 was increased in glioma tissues (Fig. 4i).

### Overexpression of KLF7 partly reverses miR-136-3p-controlled glioma cell growth, migration, and apoptosis in vitro and in vivo

**Fig. 5:**
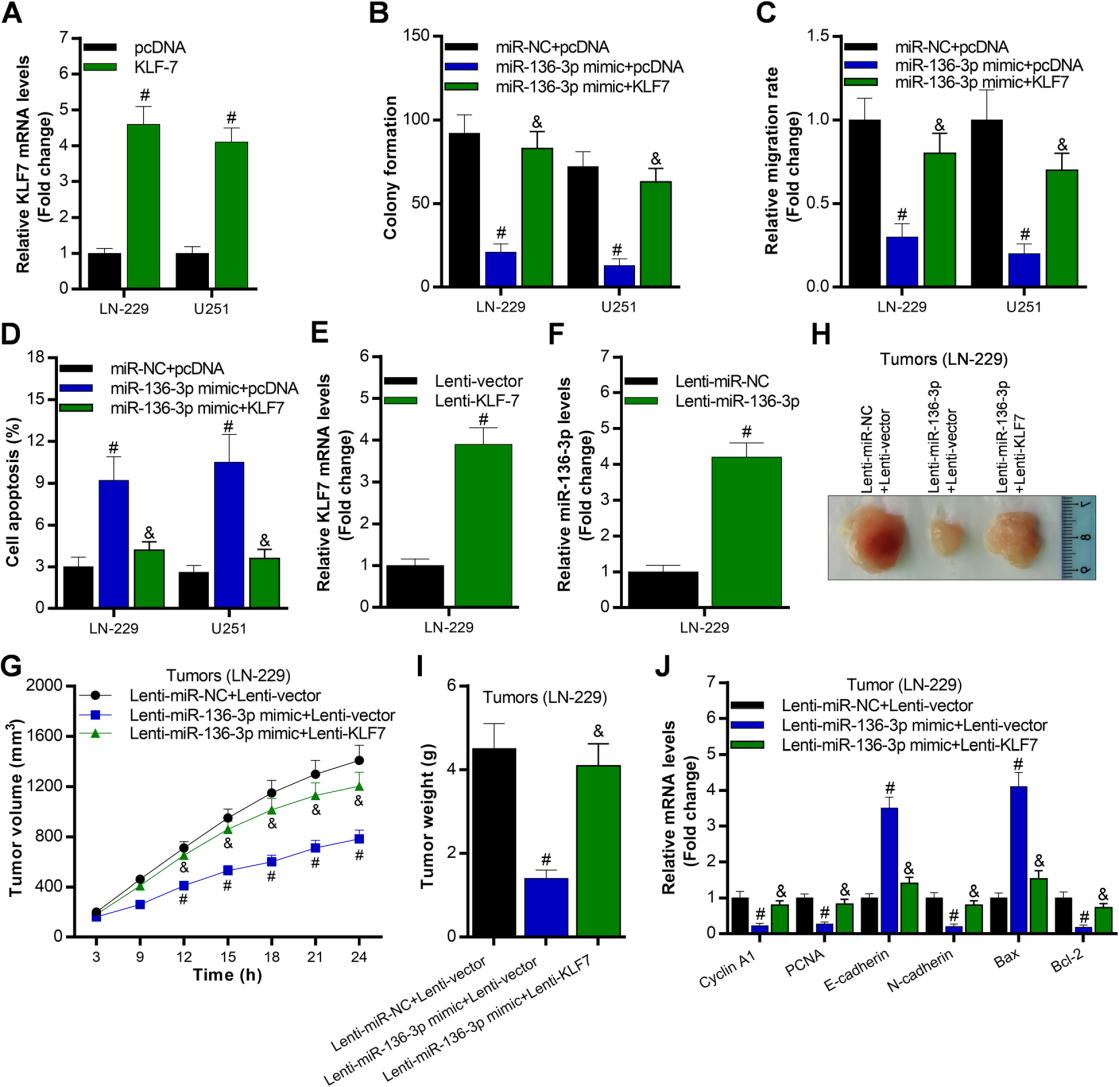
Overexpression of KLF7 partly blocks miR-136-3p-regulated glioma cell growth, migration, and apoptosis in vitro and in vivo. **a** qRT-PCR analysis of KLF7 levels in LN-229 and U251 post 48 h transfection with pcDNA and pcDNA-KLF7 (or KLF7), #*p* < 0.01, compared with pcDNA. **b** Soft-agar colony formation assay analysis of glioma cell growth after 15 days transfection with miR-NC, miR-136-3p mimic, pcDNA, or pcDNA-KLF7; #*p* < 0.01, compared with miR-NC + pcDNA, &*p* < 0.01, compared with miR-136-3p + pcDNA. **c** Trans-well analysis of glioma cell migration after 48 h transfection with miR-NC, miR-136-3p mimic, pcDNA, or pcDNA-KLF7; #*p* < 0.01, compared with miR-NC + pcDNA, &*p* < 0.01, compared with miR-136-3p + pcDNA. **d** Annexin V-FITC/PI analysis of glioma cell apoptosis after 48 h transfection with miR-NC, miR-136-3p mimic, pcDNA, or pcDNA-KLF7; #*p* < 0.01, compared with miR-NC + pcDNA, &*p* < 0.01, compared with miR-136-3p + pcDNA. **e**, **f** qRT-PCR analysis of KLF7 or miR-136-3p levels after infection with lenti-viruses for 48 h in LN-229 cells, #*p* < 0.01, compared with lenti-vector or lenti-miR-NC. **g** Tumor volume after implantation of LN-229 cells in nude mice at the different time; #*p* < 0.01, compared with Lenti-miR-NC + Lenti-vector, &*p* < 0.01, compared with Lenti-miR-136-3p + Lenti-vector. **h** The harvested tumors after 24 days implantation of LN-229 cells. **i** Tumor weight of the harvested tumors from the nude mice; Lenti-miR-NC + Lenti-vector, &*p* < 0.01, compared with Lenti-miR-136-3p + Lenti-vector. **j** qRT-PCR analysis of expression of genes, including Cyclin A1, PCNA, E-cadherin, N-cadherin, Bax, and Bcl-2 in the harvested tumors; Lenti-miR-NC + Lenti-vector, &*p* < 0.01, compared with Lenti-miR-136-3p + Lenti-vector

To determine whether KLF7 is necessarily required for function of miR-136-3p in glioma development, we co-overexpressed KLF7 and miR-136-3p in LN-229 and U251. The data showed KLF7 was successfully overexpressed by transfecting with the full length of KLF7 complementary DNA-contained plasmid (Fig. 5a). Firstly, we found that overexpression of KLF7 partly blocked miR-136-3p-regulated glioma cell growth, migration, and apoptosis of LN-229 and U251 cells (Fig. 5b, c, d). Furthermore, we also co-overexpressed KLF7 and miR-136-3p in vivo using lenti-viruses (Fig. 5e, f). Overexpression of miR-136-3p significantly decreased tumor growth indicated by tumor volume and tumor weight in nude mice (Fig. 5g, h, i). Meanwhile, overexpression of miR-136-3p obviously downregulated expression of genes, including Cyclin A1, PCNA, N-cadherin, and Bcl-2, and miR-136-3p overexpression significantly upregulated E-cadherin and Bax expression in tumors (Fig. 5j). Interestingly, we found that overexpression of KLF7 partly blocked miR-136-3p-induced suppression of tumor growth by regulating Cyclin A1, PCNA, E-cadherin, N-cadherin, Bax, and Bcl-2 (Fig. 5g- j).

## Discussion

Glioma is the most malignant tumor in brain tissues, and it has been characterized by fast infiltrative growth and dysregulated metastasis resulting in a poor overall survival rate for glioma patients [23]. Therefore, to explore molecular mechanisms could offer novel therapeutic targets for glioma treatment. Up to now, it is difficult to accurately predict prognosis for glioma patients [23]. Therefore, it is of great significance to find novel and invaluable prognostic biomarkers for glioma patients. Nowadays, increasing studies have proved that a series of miRNAs are involved in progression of multiple styles of tumors [7–13]. For instance, miR-662 [6], miR-532-5p [14], miR-199a [15], miR-940 [24], miR-200b [17], miR-34a [18], miR-130b [19], and miR-148a [20] all play an essential role in gliomas. Herein, we investigated the role of miR-136-3p and its target gene KLF7 in glioma development.

MiR-136-3p has been reported to inhibit progression of thyroid cancer [16], implying that miR-136-3p may serve as a tumor suppressor in tumors. However, little is known about the functions of miR-136-3p in other cancers, including gliomas. In this study, miR-136-3p was decreased in glioma tissues compared with the adjacent tissues. Importantly, the ability of cell growth and migration was inhibited in miR-136-3p-overexpressed cancer cells (LN-229 and U251), and the cell apoptosis was promoted in miR-136-3p-overexpressed cancer cells, suggesting that miR-136-3p may function as a tumor suppressor in gliomas. Consistently, overexpression of miR-136-3p significantly inhibited tumor growth in nude mice, conforming that miR-136-3p can serve as a cancer suppressor and it can inhibit the tumorigenesis of glioma.

MiRNAs show its function mainly by targeting 3′ UTR of mRNA in many diseases [7–9]. We predicted the target genes of miR-136-3p via using *TargetScan database.* Our results demonstrated that KLF7 was a direct target of miR-136-3p. Previous studies have revealed that KLF7 can promote polyamine biosynthesis and glioma development by activating argininosuccinate lyase [21], and meanwhile, KLF7 can be modulated by Linc00669/miR-193a axis and it can advance non-small cell lung cancer [20], suggesting that KLF7 is an oncogene. In this study, we observed that KLF7 expression was inhibited by overexpression of miR-136-3p, and KLF7 levels was increased by knockdown of miR-136-3p in cancer cells, indicating that KLF7 was a direct target of miR-136-3p in glioma. Then, in vitro and in vivo experiments were performed to determine whether KLF7 was required for miR-136-3p exerting its functions in glioma. The data showed that overexpression of KLF7 can partly block overexpression of miR-136-3p-mediated suppression of glioma progression by regulating cell growth, migration, and apoptosis of cancer cells.

## Conclusion

These findings demonstrated that miR-136-3p is significantly decreased in cancer tissues and it serves as a tumor suppressor by directly targeting KLF7 in gliomas. The results also encourage that miR-136-3p and KLF7 might be promising novel biomarkers and therapeutic targets for glioma patients’ treatment.

## Data Availability

The data and materials of this manuscript would be provided if we are required.
